# Supplementary material for: Improving the High-Frequency Response of PEI-Based Earphone with Sodium Copper Chlorophyllin
Source: Molecules. 2020 Jan 5;25(1):219. doi: 10.3390/molecules25010219 (PMC6983146; doi:10.3390/molecules25010219)
Supplement: Supplementary file 1 [file molecules-25-00219-s001.pdf]

Supporting Information

for

Improving the High-Frequency Response of PEI-based Earphone with Sodium Copper Chlorophyllin

Hao-Zhi Li, Jun-Jie Wu, Wei-Jen Lee, and Chien-Sheng Chen,*

Department of Chemistry, Fu-Jen Catholic University, New Taipei City 24205, Republic of China (Taiwan).

* To whom correspondence should be addressed:

Dr. Chien-Sheng Chen

Fax: 886-2-2653-9142

E-mail: 092847@mail.fju.edu.tw

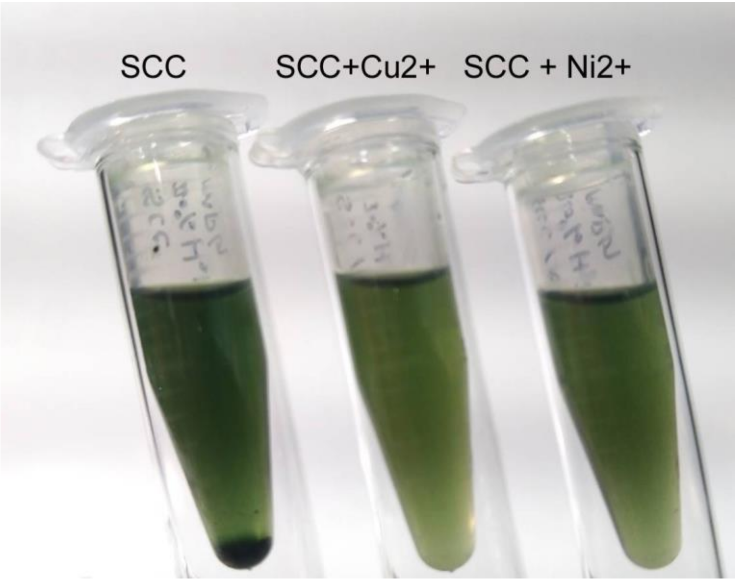

Figure S1. Solubility test of sodium copper chlorophyllin (SCC). SCC (2 mg, left), SCC/Cu(ClO_4)$_2$ (4/1, middle), and SCC/Ni(ClO_4)$_2$ (6/1, right) in MPG.

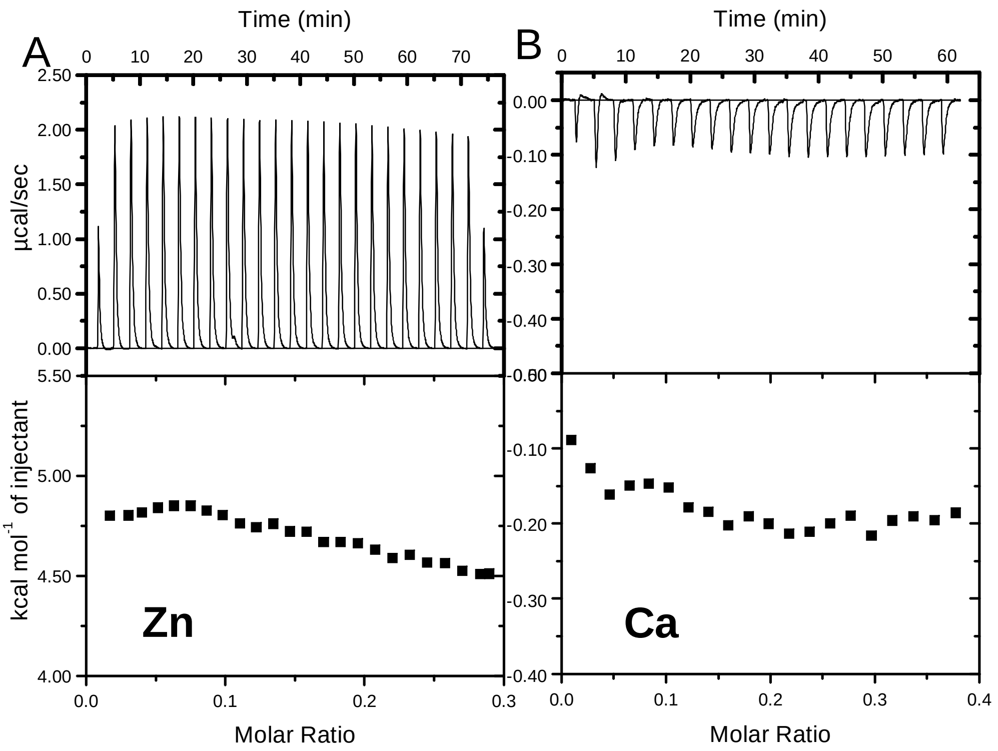

Figure S2. Binding isotherms for SCC titrated with $Zn(ClO_4)_2$ (A) and $Ca(ClO_4)_2$ (B).

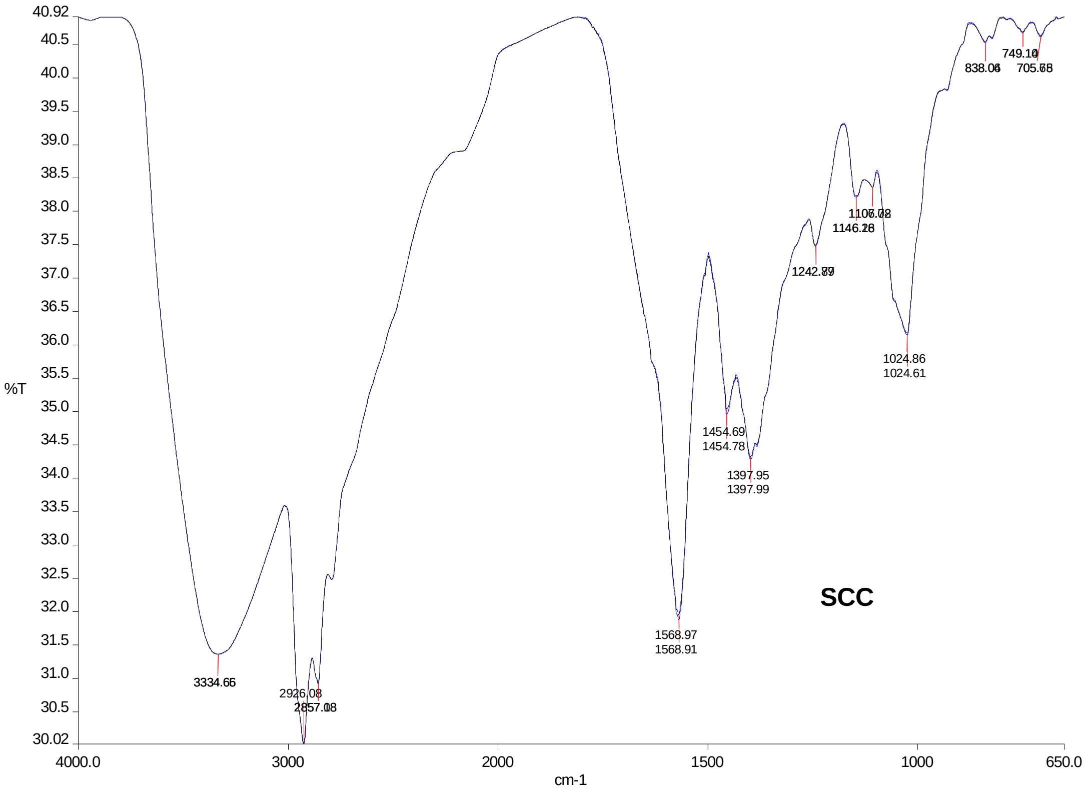

Figure S3. FT-IR spectra for SCC.

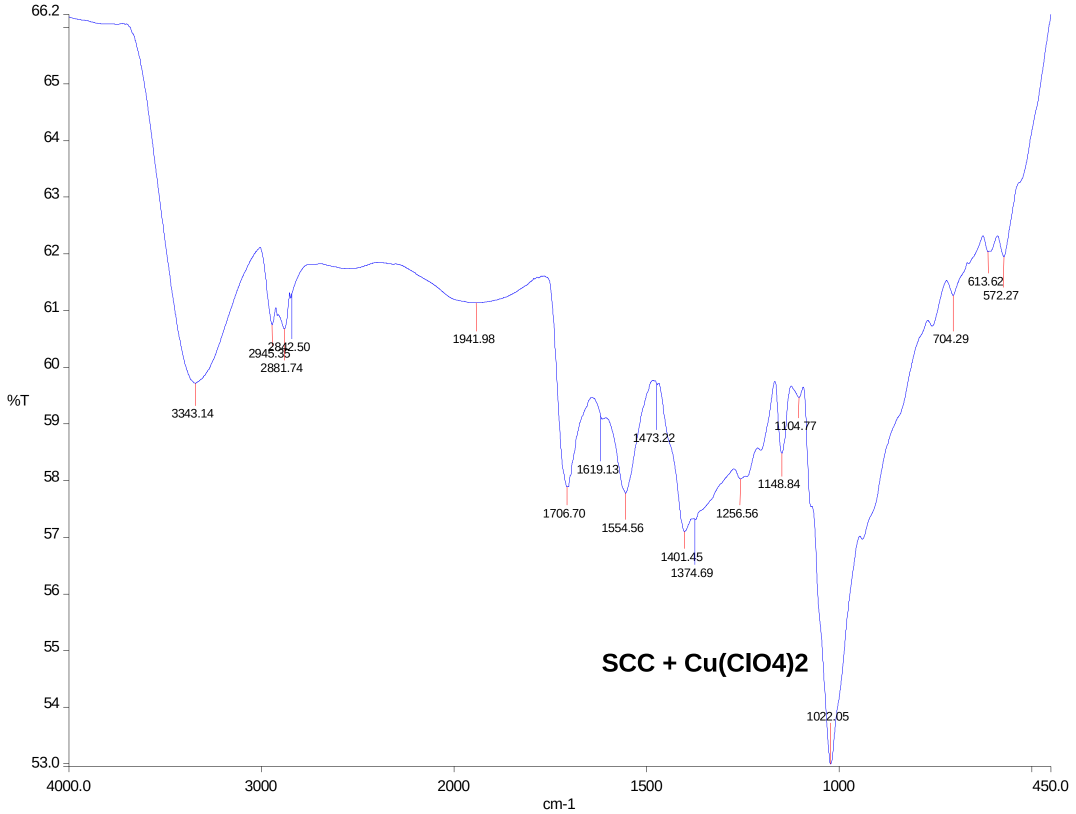

Figure S4. FT-IR spectra for $SCC/Cu(ClO_4)_2$.

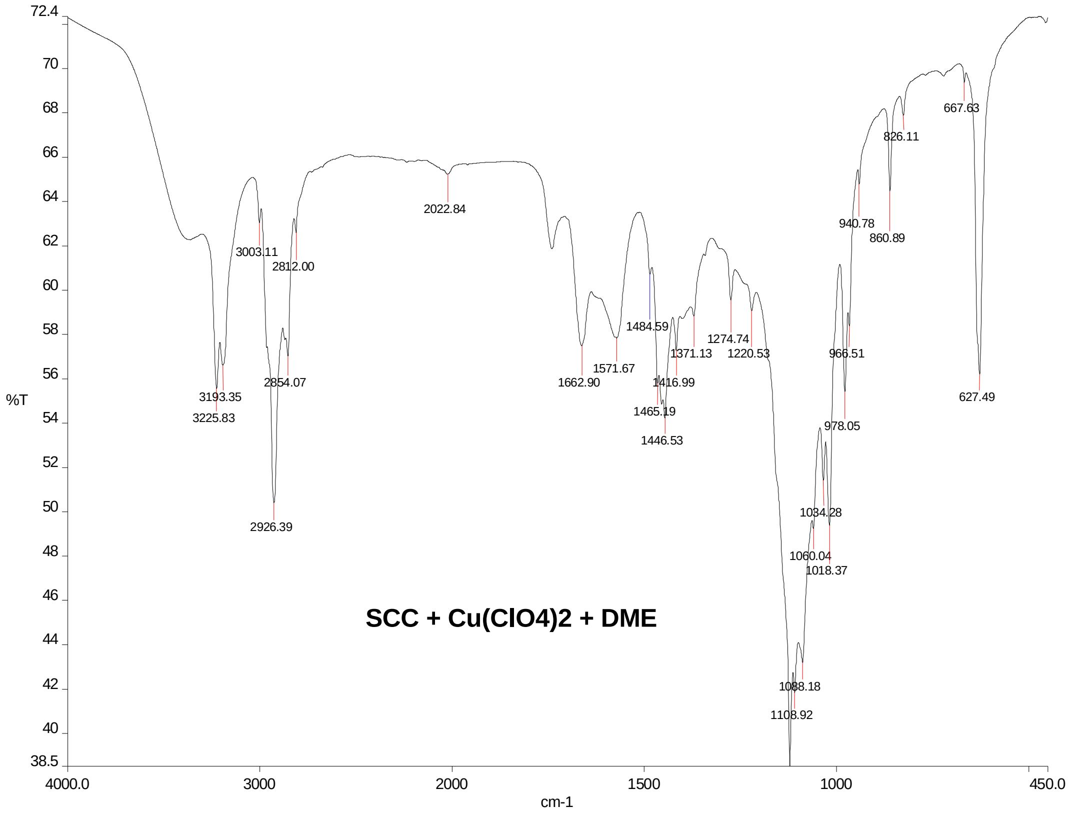

Figure S5. FT-IR spectra for SCC/Cu(ClO4)$_2$/DME.

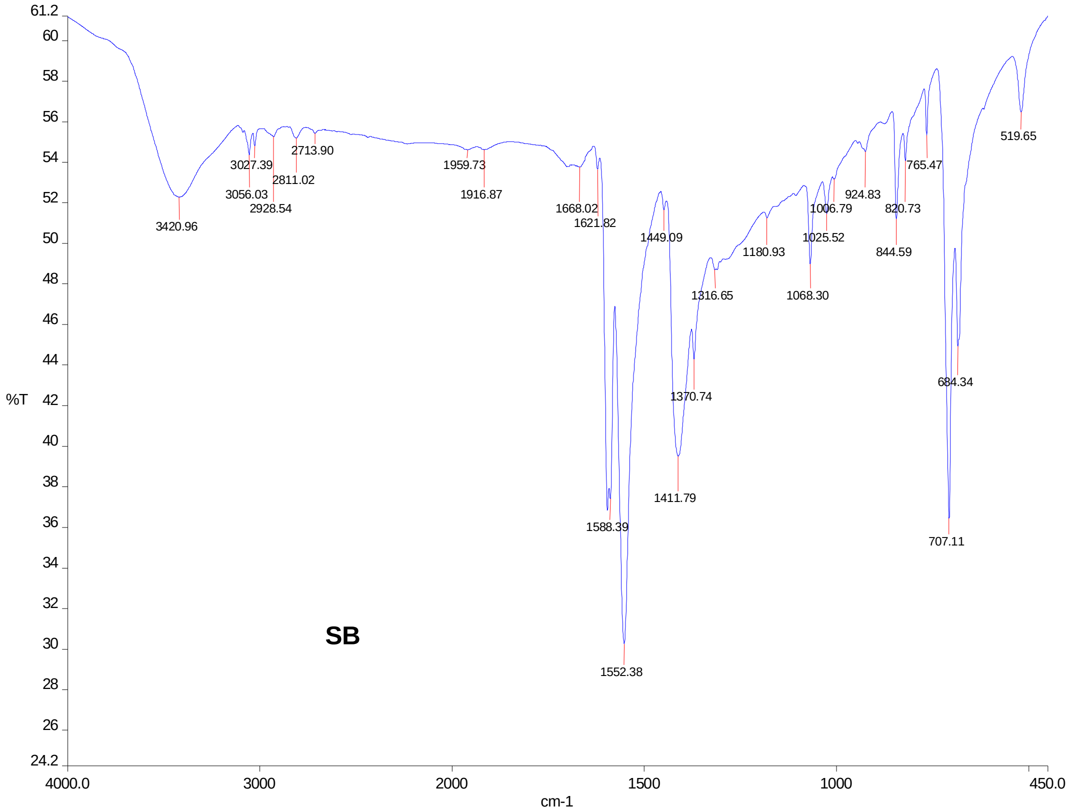

Figure S6. FT-IR spectra for SB.

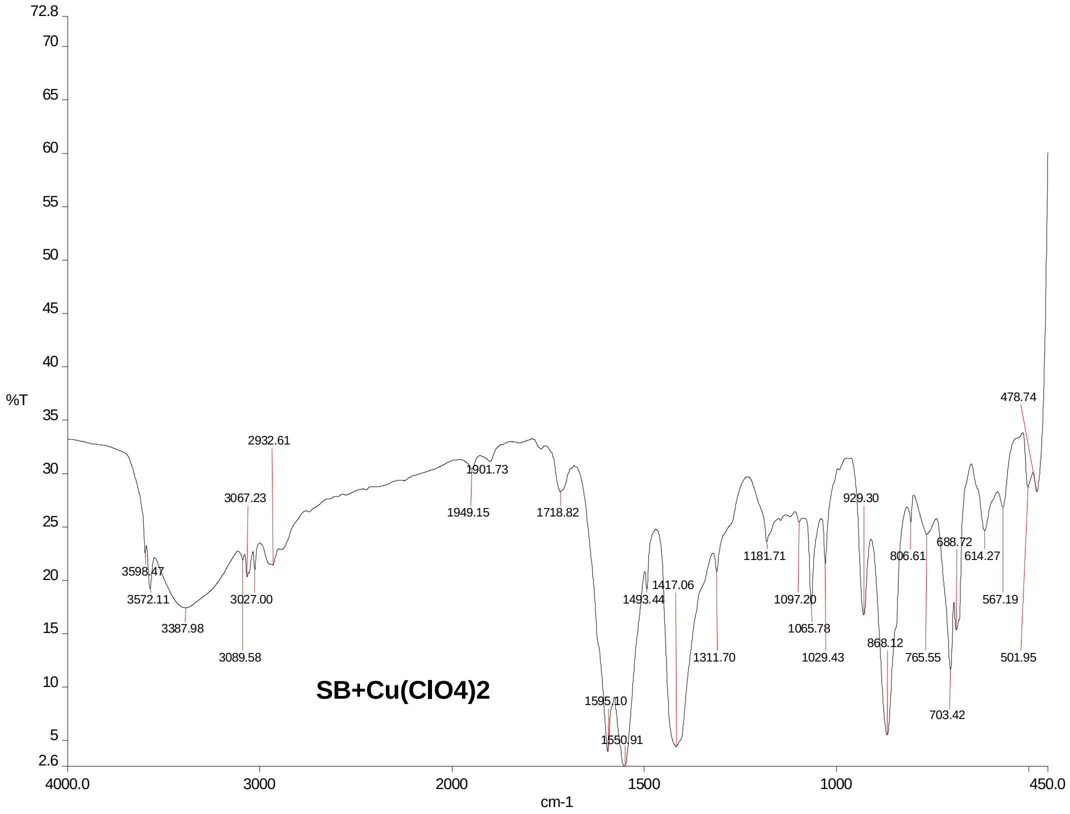

Figure S7. FT-IR spectra for SB/Cu(ClO4)$_2$.

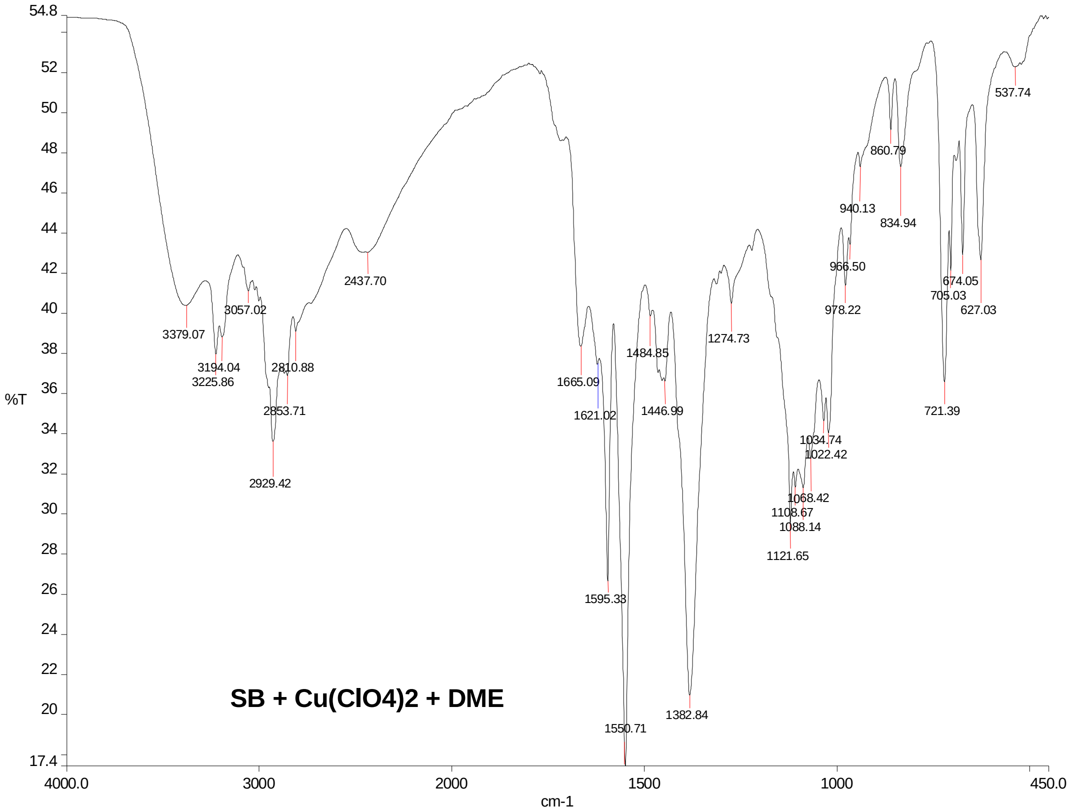

Figure S8. FT-IR spectra for SB/Cu(ClO$_4$)$_2$/DME.

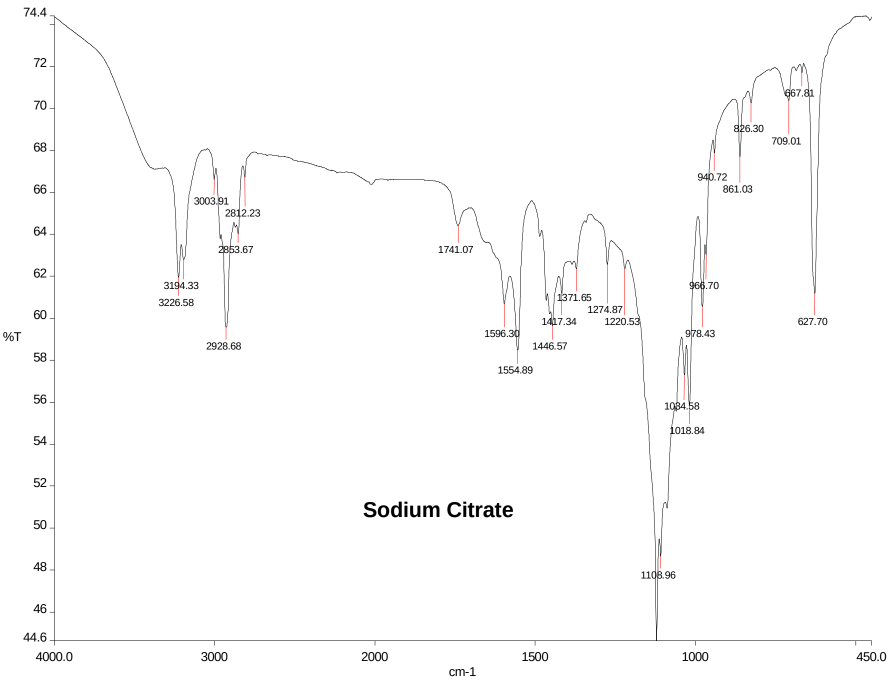

Figure S9. FT-IR spectra for sodium citrate.

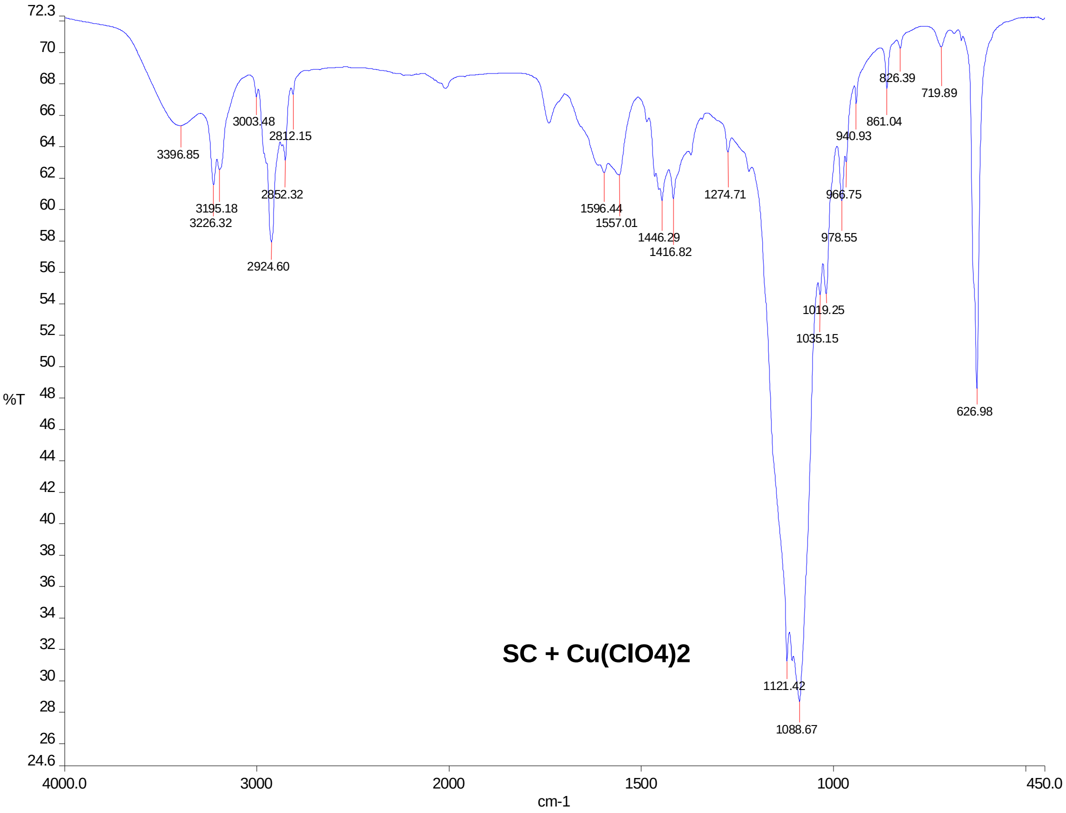

Figure S10. FT-IR spectra for $SC/Cu(ClO_4)_2$.

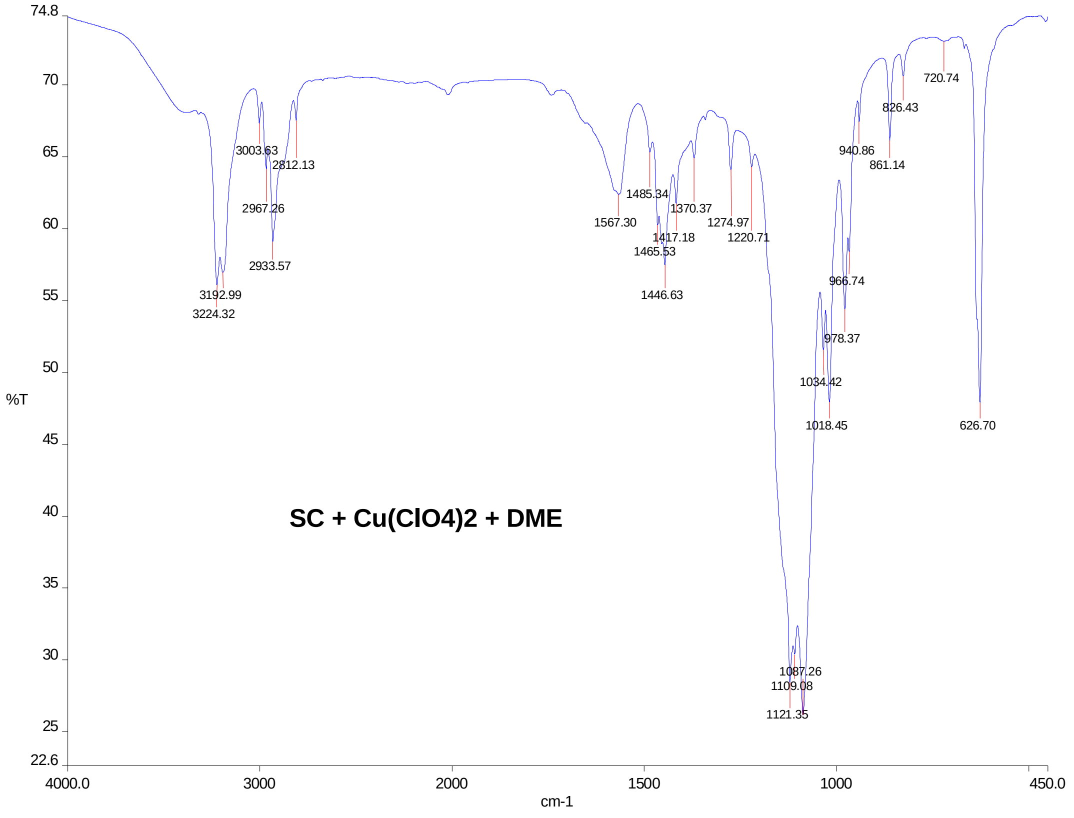

Figure S11. FT-IR spectra for SC/Cu(ClO_4)$_2$/DME.
